# Enigmatic Liaisons in Lepidoptera: A Review of Same-Sex Courtship and Copulation in Butterflies and Moths

**DOI:** 10.1673/031.012.13801

**Published:** 2012-11-26

**Authors:** Nubia Caballero-Mendieta, Carlos Cordero

**Affiliations:** ^1^ Posgrado en Ciencias Biomédicas, Instituto de Ecología, Universidad Nacional Autónoma de México, Distrito Federal, México.; ^2^ Departamento de Ecología Evolutiva, Instituto de Ecología, Universidad Nacional Autónoma de México, Distrito Federal, México.

**Keywords:** homosexuality, mating system, sexual selection

## Abstract

Same-sex sexual interactions (SSSI) have been observed in many animal groups and have intrigued evolutionists. In this paper, reports on SSSI in Lepidoptera are reviewed and evolutionary hypotheses that could explain these behaviors are discussed. SSSI have been documented in males of 25 species and in females from role-reversed populations of one species. Four types of SSSI have been reported: pupal guarding, courtship, copulation attempt, and copulation. Although the hypotheses cannot be tested with the limited data, evidence suggests that in some Lepidoptera SSSI could result from selection for imposing costs on other males, or could be a by-product of sexual selection favoring individuals that exhibit high sexual willingness. In agreement with both hypotheses, in the 17 species whose mating systems are known, there is intense competition for mates in the sex exhibiting SSSI. We propose lines of research on SSSI in Lepidoptera.

## Introduction

Numerous studies of a wide variety of insects show that courtship and copulation are costly behaviors (e.g., Dewsbury 1982; Shapiro 1982; Svard 1985; Rutowski et al. 1987; Kaitala and Wiklund 1995; Cordero 2000; Bondurianski 2001; Ferkau and Fisher 2006; Oliver and Cordero 2009). However, sometimes animals engage in same-sex sexual interactions (SSSI), behaviors that produce no offspring (Bagemihl 1999; Roughgarden 2004; Bailey and Zuk 2009). SSSI have been reported in many species pertaining to most major animal groups (Bagemihl 1999; Roughgarden 2004; Bailey and Zuk 2009). In the case of arthropods, Bagemihl (1999) summarizes published reports for 117 species in which same-sex courtship and/or copulation has been observed. Most of these species are insects (112) belonging to eight different orders, including 12 lepidopterans species (Table 1). Several evolutionary hypotheses for SSSI have been proposed, but none of them appears to explain most cases (Bailey and Zuk 2009).

This paper has two objectives. The first is to add more reports of SSSI in Lepidoptera to those listed in Bagemihl (1999). These additional reports were obtained from the literature and from personal communications. The second aim is to provide a preliminary assessment of the explanatory power of some hypotheses on the evolution of SSSI in butterflies and moths.

## Observations of same-sex sexual interactions in Lepidoptera

Observations of SSSI in 26 species of Lepidoptera are summarized in Table 1. With one exception (*Acraea encedon*; Jiggins et al. 2000), reports of SSSI were found only for males; reports on species with SSSI in both males and females were not found. Same-sex pupal guarding has been observed in the two butterfly genera known to exhibit SSSI: *Jalmenus evagoras* males gather on pupae that are close to emergence, forming “mating balls,” and the successful male copulates before the female has expanded her wings; experiments indicate that males are unable to distinguish female pupae (Elgar and Pierce 1988). Males of several *Heliconius* species perch on pupae that are about to hatch, guard them from other males, and try to mate with emerging females. In a high density captive population of *H. charitonia*, 29% of guarded pupae were males (Estrada et al. 2010).

Reports of copulation attempts commonly mention that one male approaches another male “curving the abdomen” or that, after approaching, the male “curls his abdomen,” “attempts copulation,” “attempts to mate,” or performs a “copulatory attempt.” In populations with female-biased sex ratios, *A. encedon* form role-reversed “lekking swarms” in which virgin females aggregate in areas lacking resources and solicit copulations from the very rare males (Jiggins et al. 2000). In these aggregations, “females land on top of other females when they are resting on the ground and tend to hold their abdomens curled ventrally outwards in a manner similar to that observed during mating…a typical mate acceptance behaviour…usually only seen in male-female interactions” (Jiggins et al. 2000: p. 71).

Homosexual copulations have been reported in eight species and only in males (Table 1). Copulation between females could be restricted, due to females lacking genital structures that allow effective grasping of other females (Jiggins et al. 2010).

Reports of SSSI between males of different species are shown in Table 2. Half of these interactions were between congeners, and the only interfamilial report involved three cases of a male *Thorybes pylades* (Hesperiidae) courting a “quite fresh” male *Euclidina cuspidea* (Noctuidae), a moth that flies in a “skipper-like manner.”

(Note: Cases of two males simultaneously copulating with one female have been reported in *Eucheira socialis* (Shapiro 1989), *Euphydryas chalcedona* (Masters 1974), *E. anicia* (Odendaal and Stermitz 1989–90), and *Physiodes phaon* (Perkins 1973). These cases are mentioned here because they could involve genital contact between two males.)

## Evolutionary explanations of same-sex sexual interactions in Lepidoptera

In Table 3, several evolutionary hypotheses that could explain the existence of SSSI in Lepidoptera are described (adapted from Bailey and Zuk (2009) and Stoijcović et al. (2010)). The *practice hypothesis* proposes that SSSI help improve the courtship and mating skills of sexually immature adults (as has been demonstrated in *Drosophila*; Bailey and Zuk 2009). This hypothesis predicts that SSSI will be more common in species whose adults need several days to achieve reproductive maturity, and that they will occur mainly during the pre-reproductive phase. The available information does not permit the testing of these predictions, but the fact that adults of at least some species in Table 1 are ready to mate as soon as their wings are fully extended (*Callophrys xami* (personal observation) and *Acraea encedon* (Owen 1971)) or even before (e.g., *Jalmenus evagoras* (Elgar and Pierce 1988) and *Heliconius charitonia* (Estrada et al 2010), two species exhibiting pupal mating) indicates that the practice hypothesis does not provide a general explanation for SSSI in Lepidoptera. In three species, SSSI involved recently emerged (teneral) males (Table 1), but in these cases the older (presumably sexually mature) male directed his courtship and copulation attempts to the teneral male (in the only copulation observed, it is not reported who initiated the interaction).

The *social glue hypothesis* proposes that SSSI help to establish, maintain, and improve social relationships among same sex individuals, and predicts that the incidence of SSSI will be higher in species that obtain benefits from adult gregarious behavior. Data to test this hypothesis are lacking, but it could be studied in species exhibiting adaptive gregarious roosting (such as *A. encedon* (Owen 1971), *Heliconius erato* (Salcedo 2011) and *Battus philenor* (Pegram et al. 2012)). The *indirect insemination hypothesis* proposes that SSSI permit a male to deposit sperm in another male, who then transfers it to females during heterosexual copulations. This mechanism has not been proved in any lepidopteran, and the complexity of the processes of spermatophore transfer and sperm translocation from the spermatophore to the spermatheca (see detailed descriptions and references in Drummond 1984) makes this hypothesis an unlikely explanation for SSSI in Lepidoptera.

The *intrasexual conflict hypothesis* proposes that SSSI are used to inflict damage to sexual competitors. This hypothesis predicts that a male can damage a competitor male when he actively courts, attempts copulation, or copulates with him. This damage should be expressed as a decrease in survivorship or in ability to copulate. A second prediction of this hypothesis is that SSSI will occur in species whose mating system involves intense intrasexual competition. In support of the first prediction, there is evidence that, in some species, males damage other males during homosexual “courtships” or “mating attempts” (monarchs (Rothschild 1978; Brower et al. 2007) and *Acrolepiopsis assectella* (Lecomte et al. 1988)), or when copulating (*E. editha*; Shah et al. 1986). Damage could be more likely when homosexual mating attempts and copulations are directed to fragile teneral males, as reported in three species (Table 1). The presence of spines (= cornuti) on the endophallus (Cordero 2010; Cordero and Miller 2012) or a needle-like phallus (as that of monarchs (Brower et al. 2007), *Malacosoma americanum*, and *M. disstria* (Bieman and Witter 1982)) are possible means for damaging other males during homosexual copulations. Bieman and Witter (1982) report that in male-biased populations of *Malacosoma*, males frequently attempt to mate with females in copula, and that during these attempts they sometimes pierce the abdomen of the female or her mate. In support of the second prediction, in the 17 species whose mating system is known (Table 1), the mating system is commonly associated with strong competition for mates in the sex exhibiting SSSI. In 12 species, females are polyandrous and, therefore, males experience pre-copula mate competition and sperm competition. Two species show pupal mating, a mating system in which there is strong competition for copulating with recently emerged monandrous females (Deinert et al. 1994; Elgar and Pierce 1988; Estrada et al. 2010). Furthermore, the only report of female homosexual interactions is in a butterfly, in which infection with the *Wolbachia* bacterium results in populations with heavily female-biased sex ratios that promote intense female competition for males (Jiggins et al. 2000). An idea of the intensity of competition for males in *A. encedon* is given by the fact that in one of these populations (in which the proportion of males was 0.01) 203 out of 215 females were virgin and the other 12 had only one spermatophore (Jiggins 2000). Since it seems likely that males damaging sexual competitors via SSSI also incur costs (e.g., time costs), SSSI could be a spiteful trait (West and Gardner 2010). The problem with this type of trait is that the benefits of reduced competition are also enjoyed by males not paying the costs of directing SSSI to other males. Theory predicts that this type of social action will evolve when it is directed only to nonrelatives, and indirectly benefits relatives of the actor (West and Gardner 2010). Perhaps SSSI in Lepidoptera could provide a model system to test these predictions.

The *sexual mimicry hypothesis* proposes that some individuals obtain benefits from resembling opposite-sex individuals (such as reduced harassment from dominant individuals of its own sex) and that these sex mimics receive SSSI. Considering the potentially high costs of SSSI (in terms of the risks of being damaged (see previous paragraph) and wasting time), this seems an unlikely explanation for SSSI in Lepidoptera.

The *mistaken identity hypothesis* has four versions: the first two consider that SSSI are selectively neutral or costly but maintained due to genetic constraints (i.e. maladaptive), the third version proposes that SSSI evolved due to selection favoring reduced discriminating abilities when sex discrimination is costly, and the last version considers that SSSI are a by-product of natural or sexual selection acting on some other trait (for example, when competition for mates is very intense, sexual selection could favor very high sexual responsiveness leading to sexual discrimination mistakes). The “neutral” version could apply to species in which SSSI are very rare (i.e., its costs are negligible) and do not involve damage during homosexual interactions. The “evolutionary restrictions” and “costly discrimination” versions require that SSSI are sufficiently frequent and “dangerous” as to produce fitness costs. Chaudhury and Sinha (1997) report on *Antherea mylitta* (Saturniidae) suggests that SSSI are costly because the male-male pair observed remained in copula for five days until both males died. Finally, the “sexual selection by-product” version of the mistaken identity hypothesis is a likely explanation for some of the cases of SSSI reported in Table 1. In insect species in which competition for mates is intense, sexual selection frequently favors males with a strong motivation to mate—males that continuously and actively search for mating opportunities and exhibit high sexual responsiveness (Thornhill and Alcock 1983). This strong male drive could sometimes result in discrimination mistakes and courtship and, actual or attempted, copulations with inappropriate partners (males, heterospecifics, mimicking flowers, or even inanimate objects; Thornhill and Alcock 1983), and the cases of male lepidopterans performing SSSI with males from other species seem an extreme example of this (Table 2). On the other hand, the “sexual selection by-product” version shares with the intrasexual conflict hypothesis the prediction that SSSI will occur in species whose mating system involves strong intrasexual competition. Evidence in support of this prediction was presented above.

## Concluding Remarks

The results of this survey led to the suggestion that SSSI in Lepidoptera could be more common than previously thought. The fact that in many Lepidoptera the sexes are not easily distinguished in the field suggests that SSSI could have been overlooked in several species.

The *indirect insemination* and *sexual mimicry* hypotheses appear to be unlikely explanations for SSSI in Lepidoptera, while the *practice* and *social glue* hypotheses could apply to species with particular characteristics (specifically, species in which adults of the sex exhibiting SSSI take some days to reach sexual maturity, and species that obtain benefits from adult gregariousness, respectively). Available evidence suggests that the *intrasexual conflict* and *sexual selection by-product* hypotheses are likely explanations for SSSI in several Lepidoptera.

Besides comparative studies to test the assumptions and predictions of the different hypotheses (see previous section), studies of species showing intraspecific variation in degree of intrasexual competition or in sex roles are particularly interesting to study. For example, the prediction that SSSI will be observed in the sex in which intrasexual selection is stronger could be tested experimentally in *Bicyclus anynana*, because when this species was reared at low temperatures, females showed sex role reversal and courted more frequently than males (Prudic et al. 2011), whereas when they were reared at warmer temperatures, they showed the “typical” butterfly sex-roles. Thus, it would be expected that SSSI would be observed in females reared at low temperatures and in males reared at warmer temperatures.

Finally, an interesting aspect not considered in this review is that of the proximate mechanisms resulting in SSSI in Lepidoptera. For example, young males could be perceived as females by other males if they do not produce male sex pheromone (MSP). This is the case in one-day old males of *B. anynana*, where the three presumptive components of the MSP are below detection levels (Nieberding et al. 2008). Constanzo and Monteiro (2007) found that when they experimentally blocked the structures responsible for producing MSP (androconia) in male *B. anynana*, the males were often courted by other males. This proximate mechanism is compatible with several of the evolutionary hypotheses (Table 3).

**Table 1.**
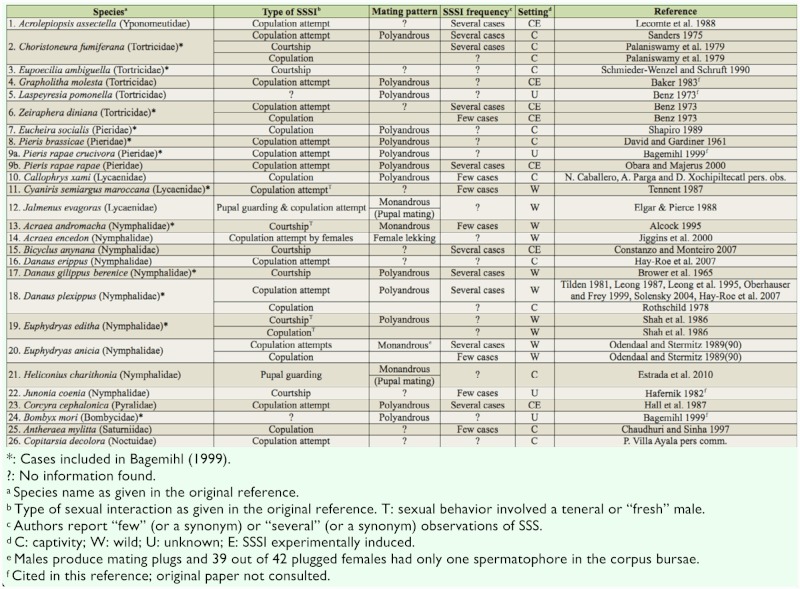
Survey of same-sex sexual interactions (SSSI) in Lepidoptera; with exception of *Acraea encedon*, all reports are of interactions between males.

**Table 2.**
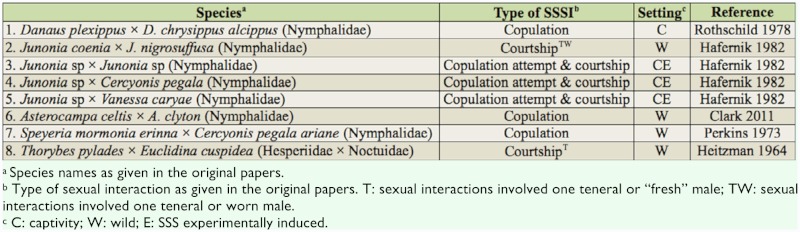
Interspecific same-sex sexual interactions (SSSI) in Lepidoptera (the first species initiated the interaction).

**Table 3.**
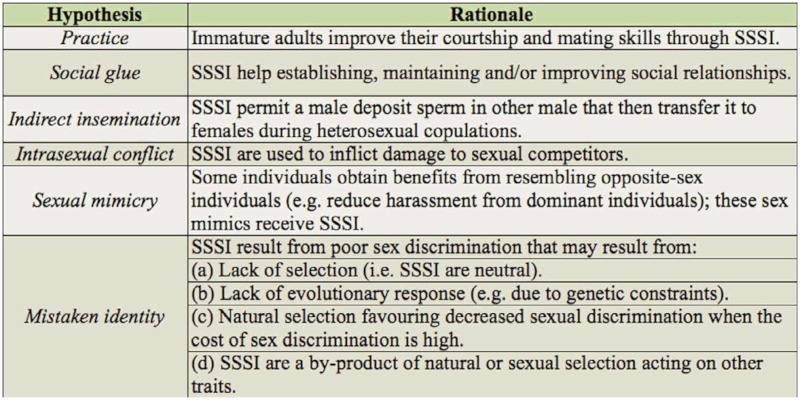
Hypotheses to explain the existence of same-sex sexual interactions (SSSI) in Lepidoptera (adapted from Bailey and Zuk (2009)) and Stoijcović et al. (2010)).

## Abbreviations

SSSI: same-sex sexual interactions
